# Graft dysfunction and rejection of lung transplant, a review on diagnosis and management

**DOI:** 10.1111/crj.13471

**Published:** 2022-01-25

**Authors:** Haishuang Sun, Mei Deng, Wenhui Chen, Min Liu, Huaping Dai, Chen Wang

**Affiliations:** ^1^ Department of Respiratory Medicine The First Hospital of Jilin University, Jilin University Changchun China; ^2^ Department of Pulmonary and Critical Care Medicine, China‐Japan Friendship Hospital； National Center for Respiratory Medicine； Institute of Respiratory Medicine Chinese Academy of Medical Sciences; National Clinical Research Center for Respiratory Diseases Beijing China; ^3^ Chinese Academy of Medical Sciences Graduate School of Peking Union Medical College Beijing China; ^4^ Department of Radiology China‐Japan Friendship Hospital Beijing China

**Keywords:** chronic lung allograft dysfunction, lung transplantation complications, primary graft dysfunction, radiological findings, treatment

## Abstract

**Introduction:**

Lung transplantation has proven to be an effective treatment option for end‐stage lung disease. However, early and late complications following transplantation remain significant causes of high mortality.

**Objectives:**

In this review, we focus on the time of onset in primary graft dysfunction and rejection complications, as well as emphasize the role of imaging manifestations and pathological features in early diagnosis, thus assisting clinicians in the early detection and treatment of posttransplant complications and improving patient quality of life and survival.

**Data source:**

We searched electronic databases such as PubMed, Web of Science, and EMBASE. We used the following search terms: lung transplantation complications, primary graft dysfunction, acute rejection, chronic lung allograft dysfunction, radiological findings, and diagnosis and treatment.

**Conclusion:**

Primary graft dysfunction, surgical complications, immune rejection, infections, and neoplasms represent major posttransplant complications. As the main posttransplant survival limitation, chronic lung allograft dysfunction has a characteristic imaging presentation; nevertheless, the clinical and imaging manifestations are often complex and overlap, so it is essential to understand the temporal evolution of these complications to narrow the differential diagnosis for early treatment to improve prognosis.

## Abstract 2

Early and late complications after lung transplantation remain essential causes of high mortality. In this review, we focus on the timing of the onset of primary graft dysfunction and rejection complications and highlight the role of imaging manifestations and clinicopathologic features in early diagnosis, thus assisting clinicians in the early detection and treatment of posttransplant complications and improving patient quality of life and survival.

## 1 INTRODUCTION

**FIGURE 1:**
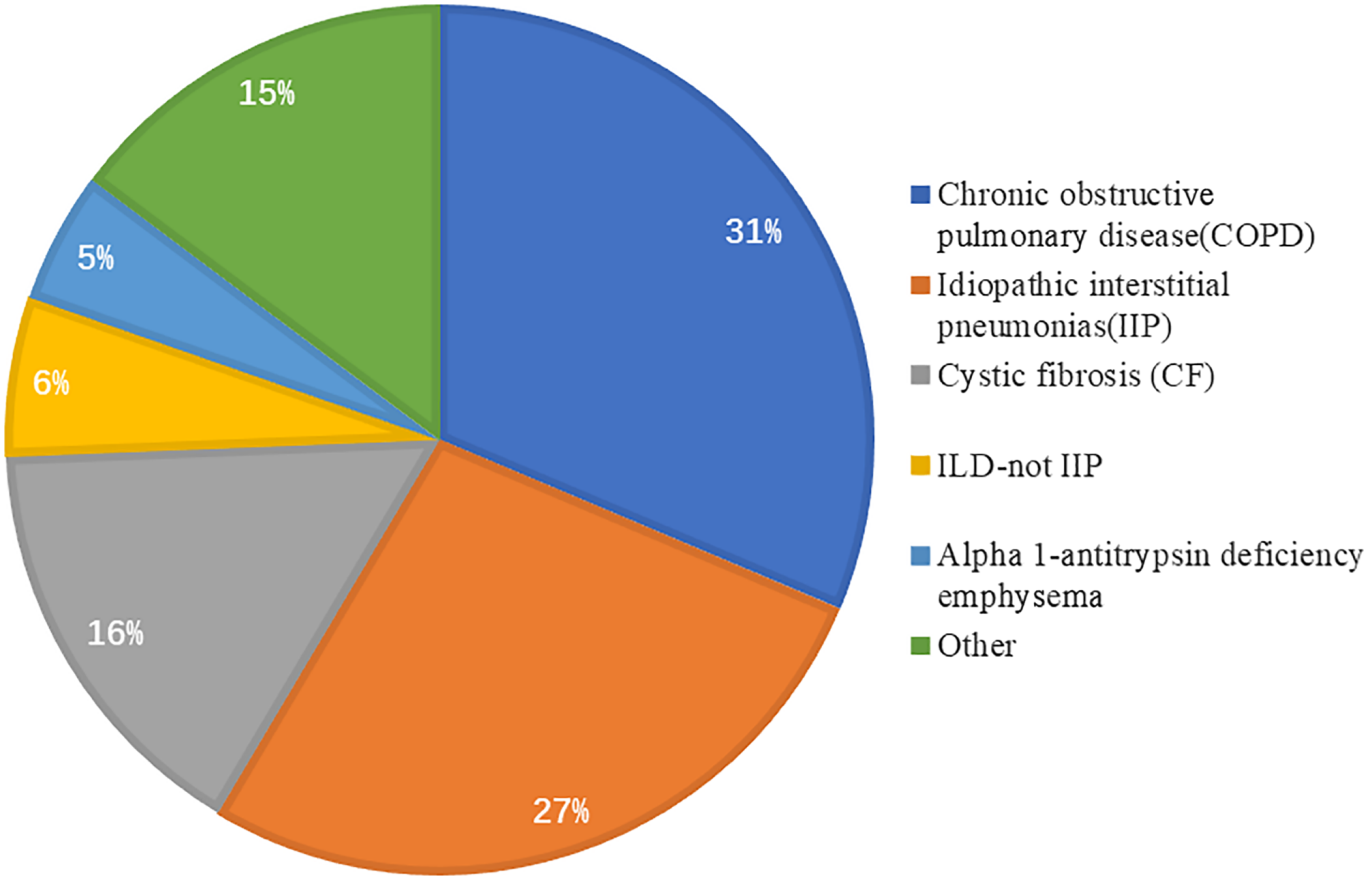
Indications for pulmonary transplantation

**FIGURE 2:**
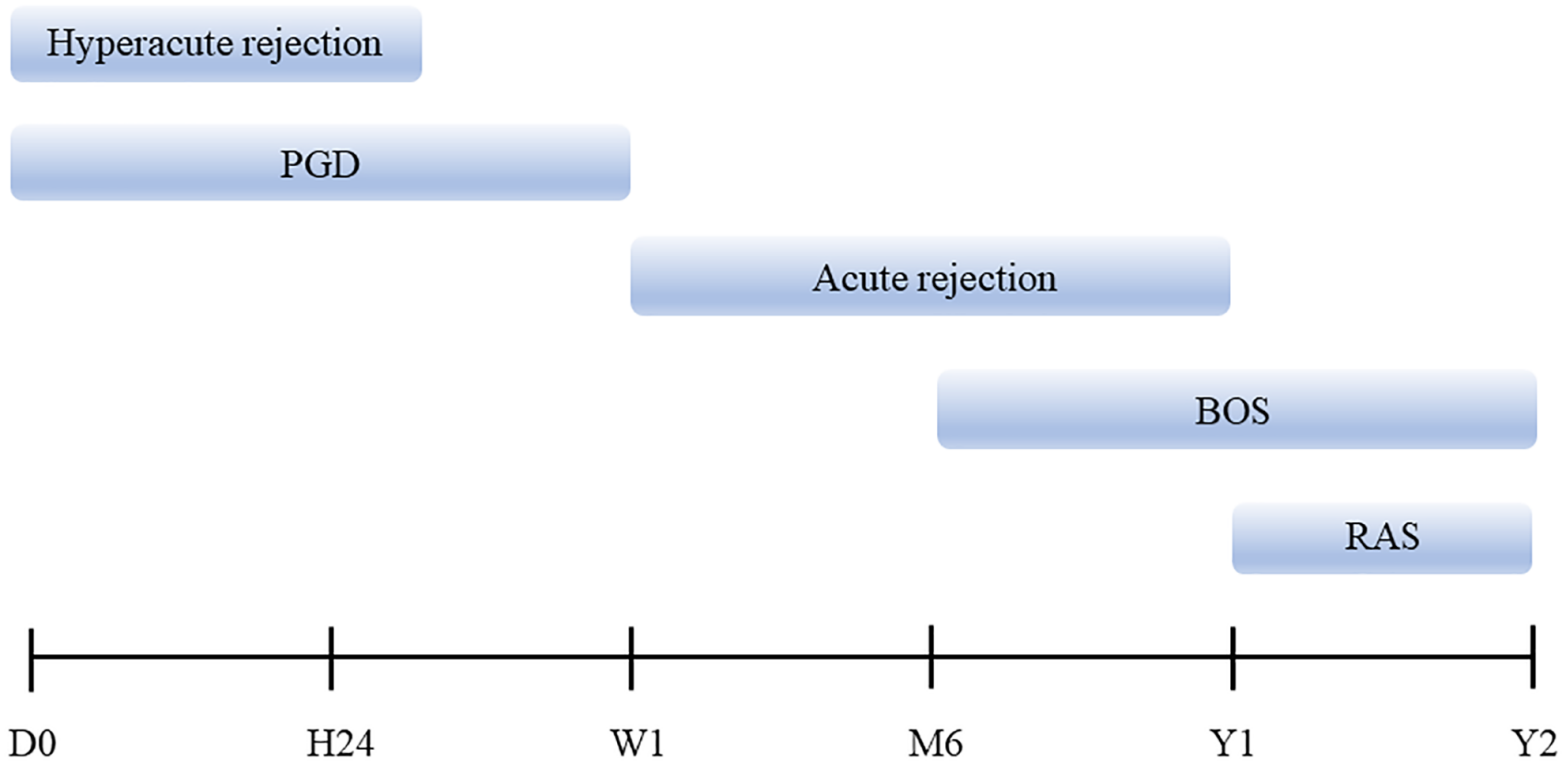
Timeline of lung transplant complications. BOS, bronchiolitis obliterans syndrome; PGD, primary graft dysfunction; RAS, restrictive allograft syndrome

There are already over 4500 lung transplants (LTs) conducted annually worldwide. Until now, more than 69 200 adult patients have received lung transplants, and the proportion of unilateral lung transplantation continues to decline, with bilateral lung transplantation becoming the predominant procedure at up to 81% in 2017.^1^ Patients with interstitial lung disease (ILD) undergoing transplantation are increasing, with idiopathic interstitial pneumonia (IIP) and non‐idiopathic interstitial pneumonia‐ILD accounting for 32.4% and 8.1%, respectively, and firstly surpass chronic obstructive pulmonary disease (COPD) as the predominant indication for adult lung transplantation in 2007 (Figure 1).^2^ However, various postoperative complications such as primary graft dysfunction (PGD), surgical complications, immune rejection, infections, and neoplasms are still unavoidable and consequently seriously affect the quality of life and survival. The 5‐year survival rate for patients undergoing lung transplantation is approximately 50%, whereas long‐term survival remains the worst of all solid organ transplants, with only 20% of 10‐year survivals.^3^ Graft dysfunction, especially chronic lung allograft dysfunction (CLAD), has evolved into a major threat to life expectancy.^4^ Therefore, early recognition and aggressive treatment of postoperative complications are extremely necessary. In this regard, radiography, especially computed tomography (CT), plays a crucial role. The atypical nature of the symptoms and the possibility of overlapping complications pose a diagnostic challenge. A list of immune complications according to chronological order is presented in Table 1. The differential diagnosis must be minimized by fully understanding the timing of complications and considering various clinical indicators. The chronology of complications after lung transplantation is presented in Figure 2. The purpose of this article is to review the risk factors, clinical, imaging, and pathological manifestations of PGD, and immune rejection complications after lung transplantation based on chronological incidence. The corresponding treatment recommendations will also be described.

**TABLE 1:** Typical signs of complications after transplantation based on chronological order

Complications	Onset	CT signs	Clinical features
Hyperacute rejection	<24 h	‐ Diffuse opacities of the graft	‐ Acute dyspnea
PGD	<1 week	‐ Basal airspace consolidations ‐ Interstitial opacities ‐ Peribronchial and intralobular septal thickening ‐ Little pleural effusion	‐ Dyspnea ‐ The ratio of P/F combined with the imaging presentation is used for PGD grading (0–3)
Acute rejection	1 week to 1 year	‐ Multifocal ground‐glass lesions ‐ Lobular septal thickening ‐ Consolidations ‐ Pleural effusion	‐ Dyspnea ‐ Cough ‐ Lower extremity edema
BOS	>6 months	‐ Air trapping and mosaic attenuation ‐ Bronchiectasis and bronchial wall thickening ‐ Tree‐in‐bud and lobular central nodules	‐ Obstruction ‐ FEV1 ≤ 80% baseline ‐ FEV1/FVC ratio <0.70
Mixed	>6 months	‐ Concurrent obstructive and restrictive pulmonary imaging signs	‐ Combined obstructive and restrictive spirometric changes
RAS	>1 year	‐ Ground‐glass opacities ‐ Apical and upper lung fibrosis ‐ Pleural thickening ‐ Traction bronchiectasis ‐ Hilar retraction and structural distortion ‐ Volume loss	‐ Restriction ‐ FEV1 ≤ 80% baseline ‐ TLC<90% baseline

Abbreviations: BOS, bronchiolitis obliterans syndrome; CT, computed tomography; FEV1, forced expiratory volume in 1 s; FVC, forced vital capacity; P/F, partial pressure of arterial oxygen (PaO2) to fraction of inspired oxygen (FiO2); PGD, primary graft dysfunction; RAS, restrictive allograft syndrome; TLC, total lung capacity.

## 2 HYPERACUTE REJECTION

Hyperacute rejection as an antibody‐mediated rejection reaction can cause acute alveolar injury.^5^, ^6^ Graft dysfunction or failure occurs during the operation or within minutes to hours postoperatively and often no more than 24 h. It is a rapidly progressive, fulminant, and fatal clinical syndrome. The preexisting antihuman leukocyte antigen (anti‐HLA) or anti‐ABO antibody in the recipient that react with the corresponding antigen present in the donor graft plays a central role in the development of hyperacute rejection. The entire transplanted lung may become edematous intraoperatively, as evidenced by sudden congestion and subsequent loss of function. On radiology, hyperacute rejection typically appears diffuse homogeneous infiltration of the entire allograft.^5^, ^7^ Although it can be treated to some extent with plasma replacement, aggressive immunosuppression, and emergency retransplantation, it is still fatal.^8^ This phenomenon becomes extremely rare with the increasing refinement of detection of sensitive reactive antibodies prior to transplantation.

## 3 PRIMARY GRAFT DYSFUNCTION

PGD is a noncardiogenic pulmonary edema and is also known as ischemia–reperfusion injury or reperfusion edema.^9^ As the most common complication during the early period after transplantation, the overall incidence of PGD is approximately 30%, with a 30‐ and 90‐day mortality of 36.4% and 23% for grade 3 PGD, respectively.^9^, ^10^ Multiple stages from preoperative donor acquisition to reperfusion can influence the occurrence of PGD. For example, preoperative donor lung ischemia, organ acquisition, preservation techniques, and intraoperative organ implantation and reperfusion are all risk factors for PGD. In addition, pneumonia and microtrauma associated with mechanical ventilation are also considered as contributing factors.^9^ The primary pathological features of PGD are ischemic pulmonary vascular injury, increased vascular permeability, and diffuse alveolar injury with characteristic hyaline membrane formation and alveolar septal thickening. It usually occurs within 72 h after transplantation, most severely at 4–5 days, and begins to subside around the first week.^11^ If it persists after the first week, infection or acute rejection should be considered. Imaging presentations are often nonspecific and variable, but most commonly are middle and lower lobes especially perihilar and basal airspace consolidations, interstitial opacities, peribronchovascular, and septal thickening but without cardiomegaly. Little pleural effusion is also visible.^12^ Definitive diagnosis often requires exclusion of other confounding etiologies such as infection, cardiogenic pulmonary edema, or rejection. If the solidity is clumpy and does not subside easily, the involvement of infection should be suspected. Apart from imaging, cardiac ultrasound can also help rule out cardiogenic pulmonary edema. Imaging and the ratio of partial pressure of arterial oxygen (PaO2) to fraction of inspired oxygen (FiO2) (P/F) are used for the grading and clinical prognostic evaluation of PGD (Table 2).^9^, ^13^, ^14^ Moreover, grade 3 PGD is strongly correlated with the decline of forced expiratory volume in 1 s (FEV1) and the development of bronchiolitis obliterans syndrome (BOS).^15^ Strict preoperative criteria for donor and recipient selection and refinement of lung perfusion preservation and surgical techniques can reduce the occurrence of PGD to some extent. Currently, PGD is still treated mainly with supportive care such as maintaining negative fluid balance, implementing protective lung ventilation strategies, and transition to retransplantation with supportive therapy and extracorporeal membrane oxygenation (ECMO) assistance for refractory PGD.

**TABLE 2:** The severity grading of PGD in ISHLT

Grade	Chest radiograph	P/F ratio
0	Normal	Any
1	Infiltration	>300
2	Infiltration	200–300
3	Infiltration	<200

Abbreviations: ISHLT, International Society for Heart and Lung Transplantation; P/F ratio, the ratio of partial pressure of arterial oxygen (PaO2) to fraction of inspired oxygen (FiO2); PGD, Primary graft dysfunction.

## 4 ACUTE ALLOGRAFT REJECTION

Acute allograft rejection after lung transplantation can occur at any time after transplantation especially with a high prevalence up to 30% of cases within the first year.^16^ The mortality rate within 30 days is about 4%. A multicenter prospective study with 400 lung transplant patients revealed that the degree of HLA mismatch related to the occurrence of acute allograft rejection and that bilateral lung transplants significantly reduced the risk of acute allograft rejection compared with single lung transplants.^17^ The clinical symptoms may include dyspnea, cough, or lower extremity edema. Till now, the gold standard of diagnosis for acute allograft rejection after lung transplantation remains transbronchial biopsy, with characteristic pathology of patchy lymphohistiocytic inflammatory infiltrate central to small blood vessels.^18^ Inflammation of the bronchioles may also be seen. The International Society for Heart and Lung Transplantation (ISHLT) has established diagnostic and grading criteria for acute allograft rejection based on the degree of lymphocytic infiltration from grade A0 to grade A4 diffuse infiltration (Table 3).^19^, ^20^, ^21^ Imaging manifestations are of poor sensitivity and specificity in acute allograft rejection, with the main signs including multifocal ground‐glass opacities, interlobular septal thickening, and consolidations, accompanied by pleural effusions or not.^22^ However, CT plays an important role in ruling out causes apart from acute allograft rejection and aiding in the localization of transbronchial biopsy. When pulmonary imaging appears normal or diffuse, biopsies are often performed from the lower lobes. And if the disease is patchily distributed on the image, then sampling is often conducted from areas of radiological abnormality.^23^ It is worth noting that despite the absence of imaging abnormalities, patients may also develop subclinical acute allograft rejection. At this time, biopsy is particularly essential for the prompt diagnosis of lesions that are difficult to observe clinically and on imaging at an early stage and therefore reduces the risk of progression of acute allograft rejection and later development of CLAD. Furthermore, significant improvement in clinical symptoms and imaging signs after early administration of high‐dose corticosteroids facilitates the diagnosis of acute allograft rejection.^24^ Immunosuppressive agents such as polyclonal antilymphocyte antibodies and interleukin 2 receptor antagonists are used immediately at the time of lung transplantation to reduce the risk of acute rejection. Additionally, hormonal shock therapy should be given in the event of acute rejection.

**TABLE 3:** Histopathological diagnosis and grading of acute graft rejection

Grade	Severity	Features
A0	None	Normal lung parenchyma without mononuclear cell infiltration
A1	Minimal	Scattered two to three layers thick cellular infiltrate around vascular
A2	Mild	More frequent dense or scattered mononuclear cell infiltrates in the perivascular area; common endotheliitis
A3	Moderate	Dense perivascular and peribronchial mononuclear cell infiltrates with interstitial involvement
A4	Severe	Diffuse infiltrate of monocytes with significant alveolar injury and endotheliitis

## 5 CHRONIC LUNG ALLOGRAFT DYSFUNCTION

**FIGURE 3:**
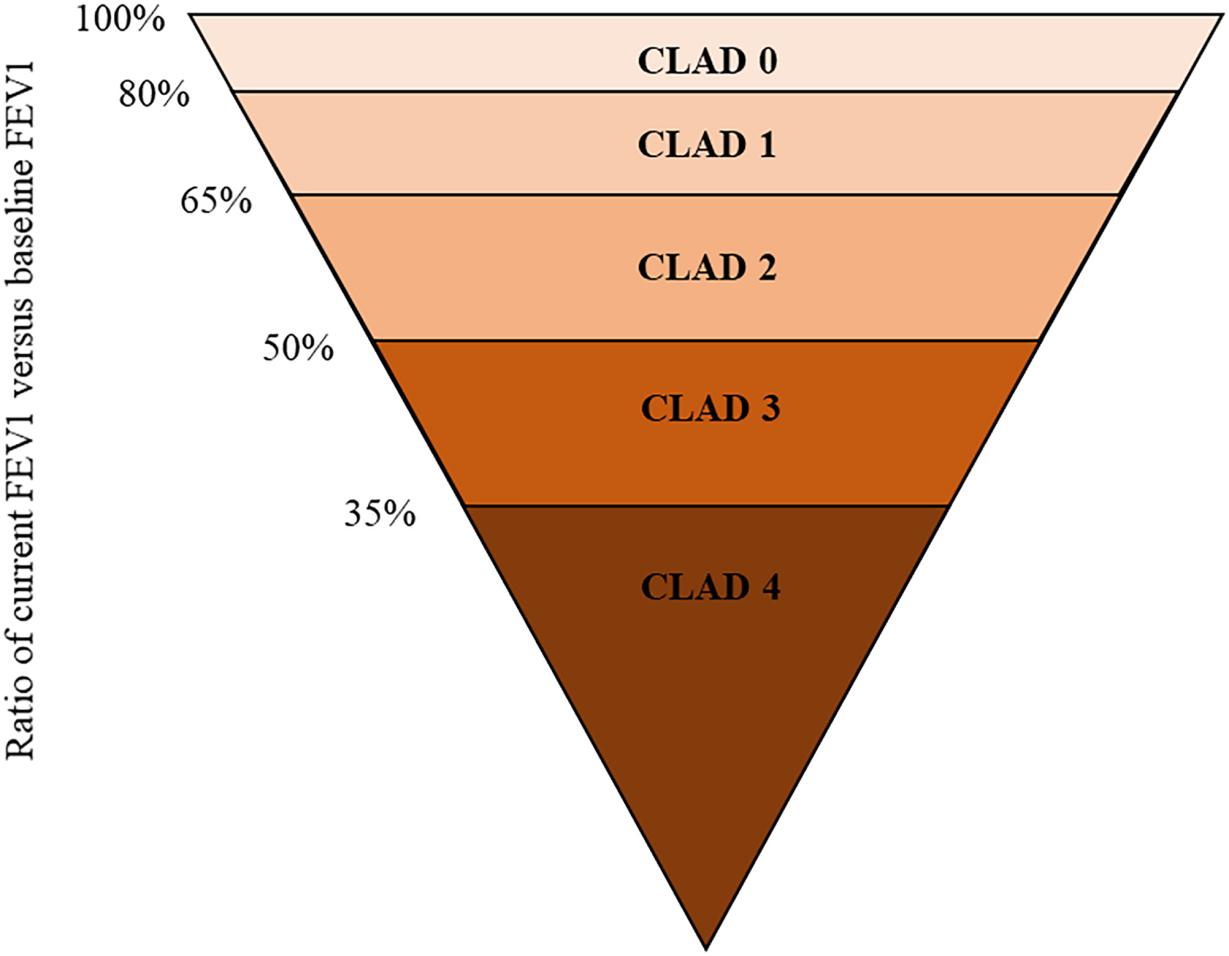
Chronic lung allograft dysfunction staging criteria. CLAD, chronic lung allograft dysfunction; FEV1, forced expiratory volume in 1 s

As a major cause of deaths after lung transplantation, CLAD is characterized by a progressive and irreversible decline in lung function, particularly a decline in FEV1 of at least 20% from baseline values.^4^ The most severe decline in FEV1 and forced vital capacity (FVC) tends to occur within 6 months after CLAD.^25^ Inflammatory factors, antibody‐mediated rejection, and fibroproliferative processes are the main mechanisms. It involves BOS with obstructive physiology, restrictive allograft syndrome (RAS) with a restrictive physiology, and mixed‐phenotype CLAD. Remarkably, BOS and RAS can mutually transform. Although clinical diagnosis of CLAD is primarily based on spirometry, CT still plays a crucial role in the diagnosis and follow‐up.^26^ CT can assist to detect the abnormalities earlier than the clinical changes. In addition, when the diagnosis of CLAD is definite, the percentage decrease in FEV1 will be used as a CLAD staging criterion (Figure 3).^4^, ^27^, ^28^

### 5.1 Bronchiolitis obliterans syndrome

BOS is considered to be the classic presentation of CLAD. The reported prevalence is 67.1% and has been the primary cause of death posttransplantation during the past 30 years.^1^ Long‐term survival of pulmonary transplantation is limited on account of BOS,^29^ with a median survival time of merely 3–5 years.^30^ BOS has been shown to affect about 10% of lung transplant recipients annually, with the highest incidence during the first 5 years after transplantation especially in retransplant cases.^31^ The pathological histology is characterized by chronic inflammation and occlusive fibrosis associated with the terminal bronchioles, with evidence of abnormal reshaping of the airway epithelium, lymphatic system, and vascular network. However, peripheral lung tissues are rarely involved.^32^, ^33^ Obstructive lesions secondary to small airway fibrosis cause intrapulmonary hyperinflation which in turn leads to increased lung volume. The main clinical symptom is respiratory distress, which is related to progressively and irreversibly obstructive spirometry decline.^34^ An FEV1/FVC ratio <0.7 with normal total lung capacity (TLC) is a typical lung function manifestation seen in BOS (Table 1). Multiple factors are involved in the development of BOS, such as air pollution, infection, airway ischemia, recurrent episodes of acute rejection, oxidative stress, exposure to airway‐specific autoantigens, and gastroesophageal reflux disease.

High‐resolution CT (HRCT) can be used as a simple noninvasive method for evaluation of BOS. Early BOS is characterized with expiratory air trapping, and in the late stages, mosaic attenuation related to vasoconstriction and insufficient blood supply due to hypoxia is common in HRCT. In addition, expiratory CT may distinguish air trapping from other causes of mosaic attenuation, such as occlusive vascular disease and infiltrative lung disease.^35^, ^36^ Moreover, when pulmonary function tests (PFTs) demonstrated obstructive CLAD, the air retention correlates with the severity of the disease.^37^ Large airway lesions, such as cylindrical bronchiectasis and bronchial wall thickening, can also be observed.^38^ The tree‐in‐bud and lobular central nodules correspond to pathologically distal bronchioles mucus embolism. However, these imaging manifestations can also be observed in acute inflammatory processes. It is necessary to exclude other confusing lesions such as infection before ascribing these imaging signs to BOS.^37^ Currently, lung retransplantation is the main treatment strategy for BOS. Early identification and intervention of BOS is helpful to delay disease progression and prolong the survival.^39^

### 5.2 Restrictive allograft syndrome

RAS accounts for approximately 35% of CLAD and is the second most common subtype of CLAD characterized by restrictive decline in pulmonary function and “stair‐step” disease progression that ultimately leads to respiratory failure and death.^40^, ^41^, ^42^ The TLC of RAS decreased ≥10% compared with the best postoperative TLC, or FVC decreased ≥20% versus the best FVC in case TLC is unavailable.^43^, ^44^ The median survival of RAS is only 6–18 months, with a significantly worse prognosis than that of BOS. It has been reported that donor‐specific antibodies (DSA) and antibody‐mediated rejection (AMR) may contribute to the development of RAS.^42^, ^45^ In addition, causes of acute lung injury such as aspiration lung injury, viral infection, and bacterial or fungal infection are also risk factors that contribute to RAS.^40^, ^46^

RAS has more complex and diverse histopathological manifestations compared with BOS.^47^ Different from BOS, the more acute diffuse alveolar damage (DAD) and intraalveolar fibrin exudation, combined with extensive irreversible end‐stage fibrosis and pleuroparenchymal fibroelastosis (PPFE), are particularly prominent.^48^, ^49^ Meanwhile, pleural fibrosis extending into the lung along the interlobular septa and fibrosis projecting from the bronchial vascular bundles both indicate a more intense fibroproliferative response to transplantation. In addition, peribronchovascular lymphocytes aggregation was accompanied by the presence of macrophages and B cells as their immune‐related presentation.^50^ Pulmonary function tests are often difficult to perform in patients with progressive RAS. Therefore, imaging features play an integral role to assist in the diagnosis of this disease.

Signs on CT may appear before the onset of restrictive ventilation disorder and may accordingly contribute to early identification and management.^51^ Furthermore, CT serves as a predictor of the occurrence and prognosis of restrictive CLAD.^26^ The presence of persistent radiographic lung opacities may be seen in RAS, whereas BOS does not have such consistent abnormal chest radiology. At the onset of CLAD, CT shows that more turbidity is often associated with the development of RAS, and there is a remarkable relation between radiological score and survival. Early RAS appears as ground‐glass opacities on HRCT, which corresponds to diffuse alveolar injury on pathology. As the disease progresses, apical and upper lung predominant fibrosis, pleural thickening, traction bronchiectasis, hilar retraction, structural distortion, and volume loss are typical imaging signs in advanced stages usually related to idiopathic PPFE. At the same time, the lung volume in advanced RAS is significantly smaller than at baseline, whereas in BOS, the lung volume is kept stable or even increased, which helps to distinguish these two subtypes.^43^ In contrast, fibrosis with inferior lung predominance is rare.^40^, ^49^ Moreover, basal predominant interstitial fibrosis and diffuse lesions have significantly worse survival versus that of predominantly apical fibrotic changes.^52^

### 5.3 Mixed‐phenotype CLAD

As the study progressed, researchers identified a third CLAD subtype with both obstructive and restrictive phenotypic features, namely, mixed‐phenotype CLAD.^53^ It can transform from one phenotype to another, and this shift often begins with BOS. In cases of previously diagnosed BOS, a decline in TLC is probably the most reliable evidence to determine the evolution from BOS to mixed‐phenotype CLAD. It can also start as a mixed phenotype. This mixed‐phenotype CLAD has multiple lesion patterns of both obstructive and restrictive phenotypes in pathology and imaging, particularly dominated apical pleural and interstitial opacities along with PPFE.^53^, ^54^

Despite efforts to differentiate CLAD using a variety of metrics, there is still a subset of cases that cannot be classified as a specific phenotype, which may be described as an undefined phenotype. Obstructive cases with persistent opacities on HRCT but without a decline in TLC or those free of opacities on HRCT but combined with both obstructive and restrictive lung function decline are all representative cases of this situation. Nevertheless, lung retransplantation remains the most effective treatment once CLAD has developed.

## 6 CONCLUSION

Being familiar with the temporal sequence of posttransplant complications and in combination with other indications (clinical presentation, pathological signs, and pulmonary function) is helpful to early diagnosis and treatment. CT can detect abnormal imaging manifestations prior to clinical symptoms and plays an essential role in early diagnosis and surveillance during follow‐up. However, these complications often have complex overlapping imaging features or frequently coexist, which poses a unique challenge for diagnosis. Additionally, by identifying the subtypes of chronic lung allograft dysfunction (CALD) and the evolution between complications, HRCT can facilitate the research on the pathogenesis of different phenotypes and the selection of subjects for clinical trials, thus ultimately enabling precise treatment. Consequently, it is extremely essential to improve prognosis by understanding the causes of posttransplantation complications, improving preoperative and postoperative management, making early diagnosis, and providing aggressive treatment.

## CONFLICT OF INTEREST

The authors have no conflicts of interest to declare.

## ETHICS STATEMENT

No formal ethical approval was needed or sought for this study.

## AUTHOR CONTRIBUTIONS

Haishuang Sun and Min Liu designed the study. Haishuang Sun and Min Liu collected the data, analyzed the data, and finalized the manuscript. Wenhui Chen participated in the collection and assembly of data. Min Liu, Huaping Dai, and Chen Wang revised the manuscript. All authors contributed to the article and approved the final version of manuscript.

## Data Availability

The data that support the findings of this study are available from the corresponding author upon reasonable request.
